# Disrupted Topological Patterns of Large-Scale Network in Conduct Disorder

**DOI:** 10.1038/srep37053

**Published:** 2016-11-14

**Authors:** Yali Jiang, Weixiang Liu, Qingsen Ming, Yidian Gao, Ren Ma, Xiaocui Zhang, Weijun Situ, Xiang Wang, Shuqiao Yao, Bingsheng Huang

**Affiliations:** 1 Medical Psychological Institute, the Second Xiangya Hospital, Central South University, Changsha, Hunan, People’s Republic of China; 2 School of Biomedical Engineering, Health Science Centre, Shenzhen University, Shenzhen, Guangdong, People’s Republic of China; 3 Guangdong Key Laboratory for Biomedical Measurements and Ultrasound Imaging, Shenzhen University, Shenzhen, Guangdong, People’s Republic of China; 4 Department of Radiology, the Second Xiangya Hospital, Central South University, Changsha, Hunan, People’s Republic of China; 5 National Technology Institute of Psychiatry, Central South University, Changsha, Hunan, People’s Republic of China; 6 Key Laboratory of Psychiatry and Mental Health of Hunan Province, Central South University, Changsha, Hunan, People’s Republic of China; 7 Shenzhen Institute of Research and Innovation, University of Hong Kong, Shenzhen, Guangdong, People’s Republic of China

## Abstract

Regional abnormalities in brain structure and function, as well as disrupted connectivity, have been found repeatedly in adolescents with conduct disorder (CD). Yet, the large-scale brain topology associated with CD is not well characterized, and little is known about the systematic neural mechanisms of CD. We employed graphic theory to investigate systematically the structural connectivity derived from cortical thickness correlation in a group of patients with CD (N = 43) and healthy controls (HCs, N = 73). Nonparametric permutation tests were applied for between-group comparisons of graphical metrics. Compared with HCs, network measures including global/local efficiency and modularity all pointed to hypo-functioning in CD, despite of preserved small-world organization in both groups. The hubs distribution is only partially overlapped with each other. These results indicate that CD is accompanied by both impaired integration and segregation patterns of brain networks, and the distribution of highly connected neural network ‘hubs’ is also distinct between groups. Such misconfiguration extends our understanding regarding how structural neural network disruptions may underlie behavioral disturbances in adolescents with CD, and potentially, implicates an aberrant cytoarchitectonic profiles in the brain of CD patients.

Conduct disorder (CD) is a fairly common neurodevelopmental disorder diagnosed in childhood and adolescence, characterized by a repetitive, persistent pattern of behavior wherein others’ basic rights and major age-appropriate norms are violated^1^. CD has been associated with genetic vulnerability, low-level physical arousal, and electrophysiological changes during feedback processing^2^,^3^,^4^ in previous researches. Neuroimaging studies have also described aberrations in brain structure and function, including prefrontal, temporal, and parietal cortical regions as well as subcortical structures, in people diagnosed with CD^5^,^6^,^7^,^8^,^9^,^10^. Most of the regions identified as having aberrations overlapped with so-called brain “hubs” in graph theory^11^, including orbitofrontal cortex, anterior cingulate cortex, insular cortex, and superior temporal cortex. These observations, which are in line with the localization of abnormalities seen in other mental disorders^12^, may indicate a crucial role of dysfunction of the corresponding complex brain networks in CD. Thus, to understand the pathophysiology of and improve clinical interventions for this disorder, it is essential to investigate the whole-brain networks of CD patients rather than focusing on an assumed regional pathology.

The notion that CD is related to abnormal brain connectivity is well supported^13^,^14^,^15^,^16^. However, primary literature has precluded adequate conclusion due to the cohort heterogeneity (with/without comorbidity), small sample sizes, and highly variable methods, and none of them investigated the large-scale structural connectivity derived from morphological correlations. Structural MRI studies have demonstrated covariance of functionally linked areas and shown that structural variance in gray matter morphology is linked with variance in physical connections formed by white matter tracts^17^,^18^. Thus, morphological correlations have been used extensively to study patterns of mammalian brain evolution^19^ and to infer structural connectivity between brain regions in humans^17^, as it provides information that complements data from other approaches and also provides crucial information that may deepen our understanding of the brain configuration.

Herein, we constructed a brain network model that accounts for correlations among regional cortical thickness values derived from each subject using graph theoretical analysis. Graph theory provides quantitative insight into network parameters governing fundamental organization of the whole brain^20^. As interest in graph theory has grown, it has been applied to studies exploring the intrinsic attributes of brain-wide architecture, such as small-worldness, efficiency, modularity, and highly connected hubs^20^,^21^,^22^,^23^,^24^,^25^,^26^. To ensure homogeneity across the two groups, we recruited forty-three CD patients (without comorbidity of attention-deficit hyperactivity disorder) and seventy-three healthy adolescents aged 13–17 in present study. The overall goal was to explore CD-related topological alterations in brain network. We hypothesized that the network-level measures (global/local efficiency and modularity) that characterize how well information is communicated within the cerebral cortex is impaired in CD patients. Secondly, the small-world architecture was assumed to be observed in CD patients as well as controls based on historical studies in other disorders^23^,^25^,^27^,^28^, with a lesser sufficiency though. Thirdly, we expected that the distribution of hubs in the CD network, which govern overall information flow, differ from that of healthy controls (HCs).

## Results

The CD group exhibited significant disruptions in both global and local brain architecture relative to HC brains, including differences in small worldness properties (σ, λ, and γ), global and local efficiency, and modularity. The CD group displayed less resilience to random failures and to targeted attacks to key regions or edges of the brain network. As we hypothesized, the hub regions derived from the CD network overlapped only partially with those of the HC network. Additionally, the group comparison of cortical thickness between the two groups was listed in supplemental materials (see Table S3).

### Changes in small-world properties

**Figure 1:**
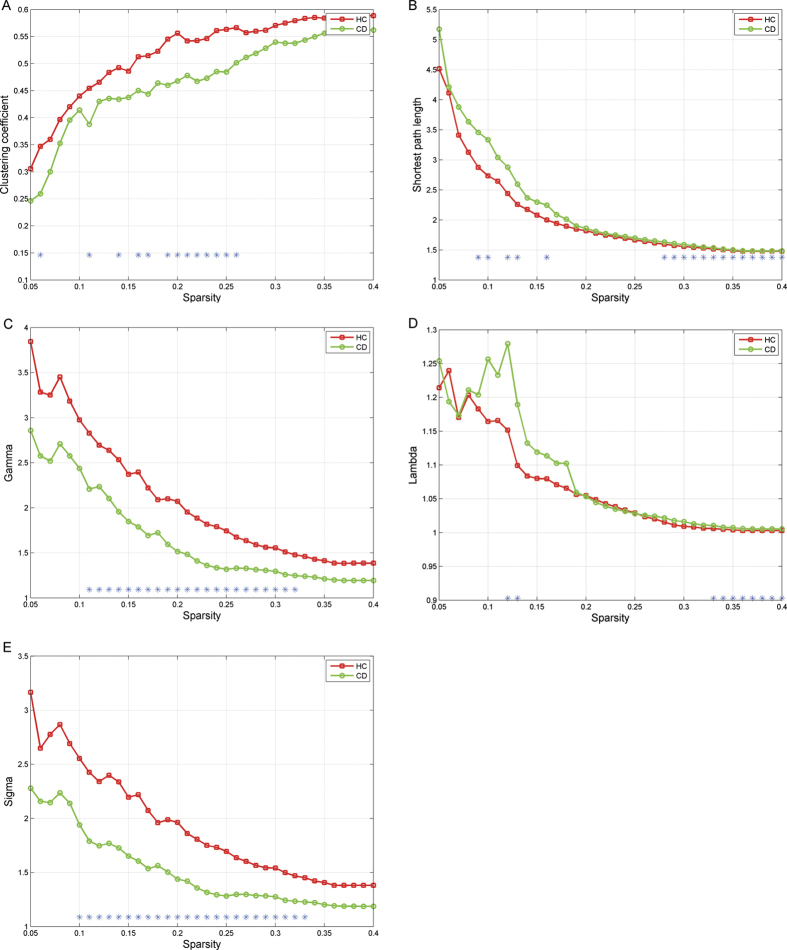
This figure indicates the comparison of five small world parameters between the two groups. (**A**) group differences in clustering coefficient (Cp); (**B**) group differences in shortest path length (Lp); (**C**) group differences in γ; (**D**) group differences in λ; (**E**) group differences in σ. *Indicates differences between groups obtained from 1000 permutation tests (P < 0.05).

Although the whole-brain networks of both the CD and HC groups demonstrated small-world network architecture with σ > 1 over a wide density range (5–40%), the CD patients had a lower γ and σ value in the 11–32% density range and 10–33% respectively, whereas the λ value showed the opposite pattern in the 33–40% density range. The CD network also had reduced areas under curves (AUCs) for γ (*p* = 0.01, uncorrected) and σ (*p* = 0.008, uncorrected) relative to the HC network. Specifically, compared to the values obtained for HCs, the CD group had a lower mean Cp (*p*_AUC_ = 0.018, uncorrected) and a higher mean Lp (*p*_AUC_ = 0.014, uncorrected) across a wide range of densities (for details, see Fig. 1).

### Group differences in network properties

**Figure 2:**
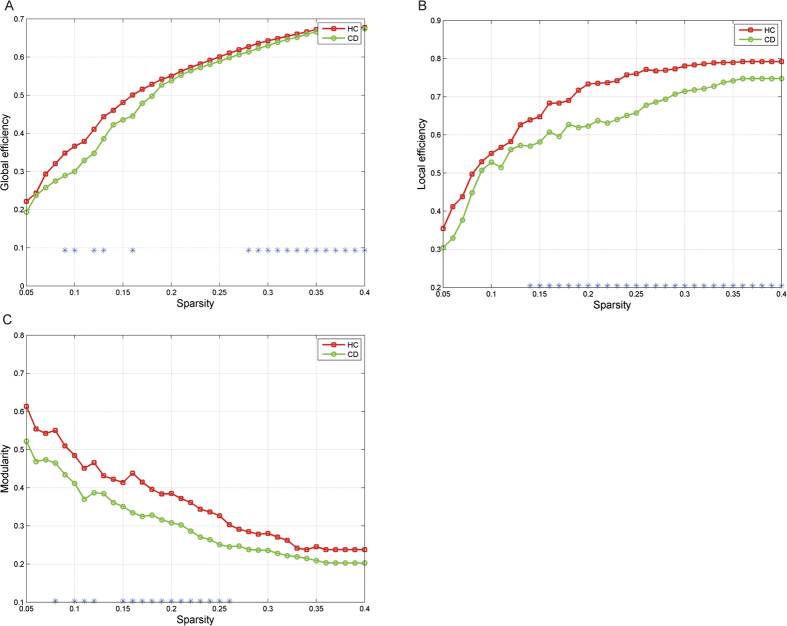
This figure indicates the group comparison of global efficiency (**A**), local efficiency (**B**) and modularity (**C**) between the two groups. CD network displayed decreased local, global efficiency as well as disrupted modularity compared with HCs across the whole densities. *Indicates differences between groups obtained from 1000 permutation tests (P < 0.05).

**Figure 3:**
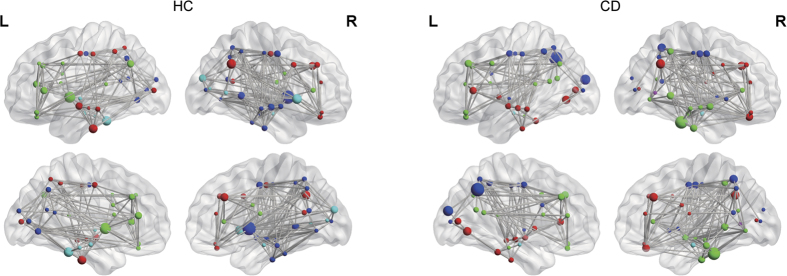
This figure displays the modular architecture of the HC (left) and CD (right) network under the density of 19%. (Red: module I, green: module II, dark blue: module III, light blue: module IV, purple: module V, respectively). The size of each node denotes the relative betweenness centrality of the cortical region in the brain network and the edges are unweighted and undirected.

The global (*p* < 0.01, bonferroni) and local (*p* < 0.01, bonferroni) efficiency AUCs for the CD network indicated impairment compared to those of HCs (Fig. 2A,B). Additionally, we observed a less optimal modular architecture in the CD group than in the HC group (*p*_AUC_ = 0.026, uncorrected, Fig. 2C, for the distribution of modules in each group, see Fig.3). Moreover, the nodal betweenness of hubs and its distributions in the two groups were quite distinct (Table 1). In CD patients, hubs were found in eight brain regions, including two paralimbic and six association areas. In HCs, hubs were located in nine areas, including three paralimbic, five association and one primary regions.

### Robustness test

**Figure 4:**
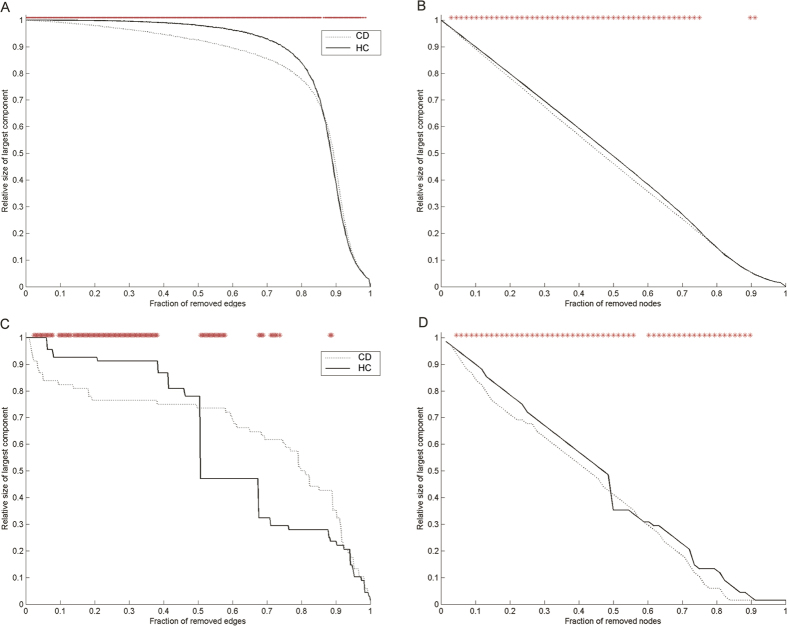
This figure illustrates the results of robustness test of CD and HC network. The top row indicates the relative size of the largest connected component (i.e., the largest subnetwork of nodes that are interconnected to each other) as a fraction of removed edges (**A**) and nodes (**B**) by random failures. The bottom row displays the relative size of the largest connected component after gradually removing edges (**C**) and nodes (**D**) according to their betweenness in a decreasing order. *indicates differences between groups obtained from 1000 permutation tests (P < 0.05).

The results of the network robustness tests, including network responses to random failures and to targeted attacks, for each group are illustrated in Fig. 4. When edges were attacked randomly, the CD connectome showed less resilience than the HC connectome (Fig. 4A). Specifically, the relative size of the largest component of the CD network began to shrink after 10% of the edges were removed, whereas the HC network did not shrink until 40% of the edges were removed. Similarly, the HC network also showed better resilience in the face of random node failures (Fig. 4B).

Targeted attacks on the most valuable 50% edges disintegrate the CD network more significantly than in the HC network. Interestingly, when more than 50% of the central edges were removed, the relative largest cluster of HC network shrank more remarkably than that of CD network, demonstrating a better resilience of the CD network than the HC network in this particular regime (Fig. 4C). When high-betweenness nodes were attacked, both networks showed rapid fragmentation, with the CD network demonstrating markedly weaker resilience to this kind of attack (Fig. 4D).

## Discussion

In this study, we used graph theory for the first time to compare the topological features of the large-scale anatomical brain networks derived from cortical thickness data from CD patients and HCs. Consistent with our hypothesis, we observed altered quantitative values of global properties in the CD network, relative to the HC network, including impaired global/local efficiency, small-world architecture, and modularity. These results, together with weaker robustness to both random and targeted attacks, suggest system-wide irregularities in global integration and segregation in the CD network. Although some of these results did not survive multiple corrections, they consistently pointed to a hypo-functioning in CD network. The present findings provide new insights into the understanding of the organizational principles of human cortical morphology in CD patients.

Although both groups exhibited small-world network attributes, the small-world topological efficiency observed in the CD network lacked the sufficiency of the HC network across a wide density range. Small-world topology is characterized by high efficiency (Cp) with a low wiring cost (Lp)^20^, and the coexistence of both attributes enables effective integration of multiple segregated sources of information across the brain^29^. Therefore, decreased Cp combined with increased Lp indicate that the information segregation and integration in CD patients are both compromised. These two processes have been associated generally with cognitive abilities^30^, such as visuo-spatial and executive functions, as well as with intelligence^31^, thus the observations in present study are in line with previous researches in which CD patients showed impairment in multiple task performances, such as those tasks requiring segregated (such as facial recognition) as well as integrated functions (such as executive function)^6^,^32^,^33^. However, we failed to infer the relationship between cognitive aspects and small world properties in present study since this structural network was constructed from a group-level and therefore, it hinders our ability to reach a more specific interpretation. Several studies have demonstrated a preserved but inferior small-world topology in rest-state network^27^,^34^ of patients, with simultaneously increased or decreased Cp and Lp, which is inconsistent with our study. Such inconsistency probably stems from a lack of standard methodological framework for the construction and characterization of brain networks between studies. Alternatively, structural covariance pattern which experiences quantitatively different developmental trajectory in relative to rest-state functional connectivity^35^, it could be shaped and refined by functional activity, and this may explain why functional networks are in place prior to structural networks^36^. Of note, the less optimal small-worldness exhibited by the CD group also provided implication for a weaker tolerance to external attacks and/or diseases^37^(discussed below).

Consistent with the prior association of more efficient connectivity of brain networks with better intellectual performance^31^, the present CD cohort showed both lower global efficiency and lower IQ scores (CD: 99.2 ± 10.0, HC 107 ± 7.2). The association might be due to common genetic factors given that topological features and intellectual capacity are both partly heritable^38^,^39^. However, there were also studies demonstrated insignificant relationship between intelligence and anatomical or functional network^27^,^40^,^41^. Such results seem not exclusive partially because of heterogeneity of methods and samples. Therefore, the relationship between IQ and brain architecture need to be elucidated in future studies which take function, resting-state and structure into account within the same cohort, yet this is beyond the scope of present study.

We also observed a diminished local efficiency in CD patients compared with HCs. Local efficiency reflects primarily the short-range connections between nearby regions that mediate fault-tolerance and modularized network information processing^42^. A higher local network efficiency yields greater fault tolerance when the network is confronted with an external attack^42^. Our findings of reduced resilience in the CD group’s network to both random and targeted network failures are consistent with this supposition.

Modularity, which allows the brain to adapt to diverse, distinct selection criteria^43^, is thought to be related to cognitive domains^44^. Therefore, disrupted modularity in CD may lead to a breakdown of information encoding between different modules that are specialized to carry out divergent tasks and may indicate a lesser capacity to adapt to multiple dynamic demands in the environment^44^,^45^, which could alter specific behavioral/emotional domains. Modularity disruptions may also provide potential markers of aberrant brain organization in relation to neurodevelopmental disorders^45^.

The identified hub regions in both groups are located primarily in association areas, with smaller hubs also being observed in limbic/paralimbic and primary regions. This observation replicates prior studies in independent samples^23^,^25^,^46^ with patients diagnosed with various conditions, such as Alzheimer disease, Schizophrenia, Epilepsy^23^,^25^,^47^. While in some resting-state and diffusion tensor imaging studies^48^,^49^, hubs were suggested to be mainly located in default-mode network (DMN), our study showed an absence of hubs in core regions of DMN, such as posterior cingulate cortex, and inferior parietal cortex. A possible explanation relies on a fact that most hubs in present study were part of one or more Rest-State Networks (RSN), and serve to link different functional modules in the brain, through partial overlap with several RSNs, such as salience network (insula), executive network (superior frontal) and visual network (cuneus)^11^,^48^. Moreover, different disorders may involve distinct sets of hubs which could reflect crucial differences in their respective pathogenic processes^12^. Notably, several network hubs observed in HCs, such as bilateral insula and the right superior temporal cortex, which showed significant group differences in betweenness, were essentially absent from the CD network. Given that selective damage in the superior temporal cortex and insula have been observed repeatedly in several CD cohorts^9^,^10^,^50^, the absence of such cortical hubs in CD may explain why certain brain regions show disproportionately high levels of connection and, as a result, preferential vulnerability to CD pathology. Since brain hubs tend to have more metabolically costly functional connections, they are likely to be more susceptible to disorders^47^.Thus, the alteration of such hubs may lead to deficient information flow and the domain of impairments characteristic of CD, such as empathy and perception of others’ feelings^51^,^52^. The relocation of hubs in CD patients, relative to HCs, on the other side, might reflect the histologically defined variations of these regions during one’s early development, since regions with high degree had greater dendritic branching of pyramidal neurons and larger-size neurons in certain area (cortical layer III), as well as more dendritic spines^53^. This is consistent with the claim that regional gray matter volume is substantially associated with regional network centrality^54^. However, this association is beyond the scope of the present study and needs to be clarified with microscopic methods.

The CD patients in this study also exhibited diminished robustness to targeted and random failures. These findings extend prior results, suggesting that networks with diminished small-worldness and efficiency tend to be less resilient to targeted and random failures^37^. According to the compensatory theory of brain functioning^55^, the relatively better organized HC network could have considerable benefits in terms of compensating for losses in network functionality in the face of external attacks^37^, such as developmental aberration or disease, during typical brain maturation. Interestingly, the HC network was more apt than the CD network to segregate into smaller pieces when almost 50% of the central edges were attacked. A possible explanation for this finding may be that due to the decreased global and local efficiency in the CD network, the peripheral edges tend to be more interconnected and thus sustained despite suboptimal communication with one another.

### Limitations

This study had some limitations. First, the non-directional structural brain networks derived from the cortical thickness values obtained for the subjects in each group constrained us from exploring correlations between behavioral or cognitive performance (such as IQ) and network parameters because the network was constructed on a group level. Secondly, we cannot say whether or to what extent the structural disruptions shown here reflect functional disruptions. Brain functional network changes dynamically over time, either in response to changing external contingencies or endogenously^34^. Thus, developmentally early functional reconfigurations might lead to structural disconnections^36^, which is in need of elucidation. Thirdly, as the network properties may unfold differently between girls and boys during adolescence^56^, the present study do not take this factor into account. Future studies need to investigate the network properties among each group separately. Finally, there is not yet a consensus regarding to the definition of structural network nodes and different parcellation strategies are known to affect connectional maps^57^. Our understanding of networks could be improved with more advanced parcellation methods, including smaller and more compact cortical partitions.

## Conclusion

The present study demonstrated topological structural changes, both local and long-range, in CD patients. Given that graph measurement value alterations can accompany suboptimal cognitive capacities, disrupted structural organization which may reflect aberrant functional reconfigurations in CD patients, could impair higher-order cognitive functions that demand access to large, integrated neuronal workspaces. Thus, to consolidate the foundation of findings observed in present structural network, it is essential to elucidate the macroscopic mechanisms by incorporating multi-modal data with network models of the connectome and more importantly, exploring underlying cytoarchitectonic profile from microscopic perspective.

## Methods

### Subjects

Forty-three adolescent patients with CD were recruited from outpatient clinics affiliated with the Second Xiangya Hospital of Central South University (Changsha, Hunan, China). A diagnosis of CD was determined based on results from the Structured Clinical Interview for the DSM-IV-TR Axis I Disorder-Patient Edition (SCID-I/P) by two well-trained psychiatrists^58^. To improve the reliability of the diagnostic interview, information was collected from each participant and at least one corresponding parent. A psychiatrist made the final decision if the information offered was inconsistent.

A group of HC subjects was selected randomly from local middle schools in the Changsha region. They were also subjected to the SCID-I/P by the same group of psychiatrists that evaluated the CD group. None of the controls met the criteria for CD or any other psychiatric disorders, or had a history of CD symptoms or aggression. Finally, seventy-three healthy adolescents composed the HC group.

The exclusion criteria were prior diagnoses of attention-deficit/hyperactivity disorder, oppositional defiant disorder, or any psychiatric or emotional disorder; diagnoses of any pervasive developmental or chronic neurological disorder, Tourette’s syndrome, post-traumatic stress disorder, obsessive compulsive disorder; a history of persistent headaches, head trauma, alcohol or substance abuse over the past year; contraindications to MRI, or an IQ ≤ 80 on the Chinese version of the Wechsler Intelligence Scale for Children (C-WISC). The C-WISC was used to measure IQ of subjects in both HC and CD groups^59^. Participants were also required to be right-handed, according to the Edinburgh handedness inventory^60^.

The study was conducted in accordance with the Declaration of Helsinki and was approved by the Ethics Committee of the Second Xiangya Hospital of Central South University (No: CSMC-2009S167). All subjects and their parents were informed of study’s purpose and written informed consent was obtained from all participants and their parents. The participants’ demographic characteristics are summarized in supplementary Table S1.

### Image acquisition and processing

We obtained three-dimensional (3D) T1-weighted images (Philips, Achieva, 3.0 T, Netherlands) for all participants’ brains using the following scan parameters: repetition time = 8.5 ms, echo time = 3.743 ms, flip angle = 8°, number of slices = 180, slice thickness = 1.0 mm, matrix = 256 × 256, reconstructed image voxel size = 1.0 × 1.0 × 1.0 mm^3^.

Anatomic reconstruction of the cortical surfaces was performed with the FreeSurfer image analysis suite (version stable 5.3.0; http://surfer.nmr.mgh.harvard.edu) as described previously^61^,^62^. Visual inspection by raters unaware of the clinical diagnosis was performed to correct errors due to miss-classification manually. An automatic parcellation technique was used to subdivide each hemisphere into 34 labeled gyral components^63^, (node labels see supplemental Table S2), with each representing a node in the network. After reconstructing the gray matter surfaces, we computed the cortical thickness of each node in all subjects.

### Network construction

We constructed the network by computing Pearson correlations between each pair of nodes^24^,^64^, and then the resultant correlation matrix (68 × 68) for each group was obtained (see supplementary Fig. S1). We included only positive supra-threshold correlations in the network based on observation that only positive morphometric correlations are mediated by direct fiber pathways^18^. The positive values of the correlation coefficient matrix R were thresholded into a binarized connectivity matrix A, where an entry *a*_ij_ equals 1 if *r*_ij_ exceeded a given threshold and 0 otherwise (see supplementary Fig. S2). Nonzero entries in the matrix corresponded to inter-regional connections. The resultant binarized 68 × 68 matrix was thus equivalent to an undirected network with 68 nodes (i.e., brain regions) and K edges, where K is the total number of nonzero elements in the matrix when diagonal elements in the matrix are set to 0. Network density was defined as the percentage of the total number of connections, K, divided by the number of possible connections as follows: density = K/(68 × 67) × 100%.

To quantitate structural cortical network properties, we employed the small-world network parameters (clustering coefficient Cp, characteristic path length Lp, and γ, λ, and σ) proposed by Watts *et al.*^65^ across a wide density range (5–40%) at an interval of 0.01^23^,^25^. This range was chosen to allow small-world network properties to be estimated adequately and the number of spurious edges in each network to be minimized as described previously^46^,^66^,^67^,^68^. A small-world network, characterized by short path lengths between individual regions and a high degree of clustering^65^ is an architecture that ensures both efficiency of specialization and integration of distributed networks^29^.

We also investigated efficiency and modularity within the brain^42^,^69^,^70^. Efficiency of a network measures how efficiently it exchanges information. Employing this simple measure, small-world networks emerge as systems that are both globally and locally efficient. Modular architecture, which represents how well functionally specialized subsystems are segregated from each other in a network^69^, is composed of groups of tightly connected cortical regions, wherein nodes within the same module have relatively stronger links and nodes in different modules have relatively more sparse connections (for calculations of efficiency and modularity Q, see the supplementary materials).

Hubs have been described as way stations for network traffic and possess a key role in interregional communication^37^. On a nodal-level, we assessed the “betweenness centrality” of each network node (see supplementary material). Hubs with high betweenness were defined as nodes whose betweenness was more than two times the average betweenness of the network, as described previously^25^.

For detailed descriptions of the uses and interpretations of these network measures, see review by Rubinov, M.^71^; the relevant formulas are also provided in Supplementary materials.

### Network robustness analysis

Network robustness, characterized by the extent of tolerance against random failures (including both nodes and edges) and targeted attacks, is associated generally with the stability of a complex network^72^. We conducted random failure analyses in which randomly chosen nodes or edges of a network in each group were removed independent of their betweenness, and then we computed the relative size (the largest sub-network of nodes that are mutually reachable/all 68 nodes of this network) of the remaining largest connected components as described previously^23^. We performed each random process 1000 times, and the mean relative size of the largest connected component was computed. Additionally, we conducted targeted attack analyses wherein we removed nodes or edges in decreasing order of their betweenness and computed the relative size of the largest component. Group comparisons of robustness-test results were performed after thresholding the correlation matrices of HC and CD groups at a fixed network density (19%), which ensured that each network had the same number of edges or wiring cost across both groups^46^ and that the between-group differences reflected alterations in topological organization rather than differences in low-level correlations^25^.

### Statistics

Before comparing the network parameters, we compared the group differences in cortical thickness. Differences in network parameters (Cp, Lp, σ, λ, and γ, modularity, and global/local efficiency, nodal betwenness of hubs) as well as network robustness measures were assessed separately for each group and compared by nonparametric permutation tests with 1000 repetitions^73^. In each randomization procedure, cortical thickness data from each subject were reassigned randomly to one of the groups. We then obtained connectivity matrices, network parameters, and network robustness measures in each randomized group and calculated their differences. Similar to previous work^66^, this analysis (except for the robustness test and hubs betweenness, which were computed and compared based on a network sparsity of 19%) was replicated across a density range (5–40%). To quantify the scalar topological network features independent of single threshold selection, we calculated the area under the curve (AUC) for each network metric and corrected for multiple comparisons. The resultant data were used to develop the permutation distribution of differences under the null hypothesis. The true between-group difference was then placed in its corresponding permutation distribution, such that its percentile rank yielded a significance level for a 2-tailed group difference between CD subjects and HC (significance threshold, *p* < 0.05).

## Additional Information

**How to cite this article**: Jiang, Y. *et al.* Disrupted Topological Patterns of Large-Scale Network in Conduct Disorder. *Sci. Rep.* **6**, 37053; doi: 10.1038/srep37053 (2016).

**Publisher's note:** Springer Nature remains neutral with regard to jurisdictional claims in published maps and institutional affiliations.

## Supplementary Material

Supplementary Information

## Figures and Tables

**Table 1:** Regions showing high betweenness (Hubs) relative to random networks for each group.

Region	Class	NB	P value
*HC group*			
Right insula	Paralimbic	5.34	<0.001*
Left insula	Paralimbic	4.16	0.001*
Right transverse temporal	Primary	3.30	<0.001*
Left fusiform	Association	3.18	0.019*
Right pars triangularis	Paralimbic	3.15	0.306
Left inferior temporal	Association	2.86	0.007*
Right superior frontal	Association	2.42	0.299
Right cuneus	Association	2.15	0.004*
Right superior temporal	Association	2.14	0.423
*CD group*			
Left precuneus	Association	4.84	<0.001*
Right fusiform	Association	4.41	<0.001*
Left cuneus	Association	3.19	<0.001*
Right pars triangularis	Paralimbic	2.75	0.306
Right superior frontal	Association	2.46	0.299
Left lingual	Association	2.37	0.166
Right superior parietal	Association	2.36	0.001*
Right medial OFC	Paralimbic	2.02	0.027*

NB, Normalized Nodal betweenness; HC, healthy control; CD, conduct disorder; OFC, orbitofrontal cortex. P values indicate differences between groups obtained from 1000 permutation tests (under a network density of 19%).
