# Supplementary material for: Disrupted Topological Patterns of Large-Scale Network in Conduct Disorder
Source: Sci Rep. 2016 Nov 14;6:37053. doi: 10.1038/srep37053 (PMC5107936; doi:10.1038/srep37053)
Supplement: Supplementary Information [file srep37053-s1.doc]

**Disrupted Topological Patterns of Large-Scale Network in Conduct Disorder**

Yali Jiang, Weixiang Liu, Qingsen Ming, Yidian Gao, Ren Ma, Xiaocui Zhang, Weijun Situ, Xiang Wang, Shuqiao Yao, Bingsheng Huang.

**Supplemental Methods and Materials**

**Definition of Network Parameters**

***Small-world properties*** To perform a graph theoretical analysis, we describe the obtained structural connection matrix as an undirected graph G with N nodes and K edges, where nodes represent brain regions and edges represent undirected connections between regions. In this study, we estimated small-world metrics of the graph G according to [^1^](#_ENREF_1), including the clustering coefficient Cp, the characteristic path length Lp, and γ, λ and σ.

Cp is a measure of the extent of local cluster or cliquishness of the network. By definition, the Cp of node i is the number of connections between the node (i)'s neighbors divided by all its possible connections, it is computed as:

Cp_i_ =

While E_i_ is the actual number of connections among the neighbors of node i, and k_i_ is the degree of node i (i.e., total number of neighbors), the k_i_ (k_i_–1)/2 means the number of all possible connections among the neighboring nodes. Obviously, 0 < Cp < 1; and only in a fully connected network, that is, each node is connected to all other nodes, Cp = 1. The Cp of the graph G is an average Cp of all nodes within the network.

Cp = $\frac{1}{n}\sum_{i-1}^{n} ci$;

The mean shortest path length Lp (i) of a node i was defined as the average of the shortest path lengths between this node and all other nodes in this network.

Lp_i_ = ;

The Lp of the graph G is averaged overall all pairs of nodes.

Lp = ;

Lp is a measure of quantifying how efficiently information can be transmitted from one node to the rest of the network. Of note, characteristic path length was measured by the harmonic mean distance between all possible pairs of regions to overcome the problem of possibly disconnected network components as previous work .

The characterization of small worldness can also be summarized into three simple quantitative measurements :

γ = λ = σ =$\frac{\text{γ}}{\text{λ}}$

While the values of Creal and Lreal of the anatomical network were compared with those of 1000 random networks which preserves the same number of nodes, mean degree , and degree distribution as the real network [^5^](#_ENREF_5). Typically, a small-world network should fulfill the following conditions:

γ = >>1 ; λ = ; σ =$\frac{\text{γ}}{\text{λ}}$ >1.

***Efficiency*** For a graph (network) G with N nodes and K edges, the global efficiency of G can be computed as [^6^](#_ENREF_6):

E_glob_(G) = ;

E_loc_(G) = .

While d_ij_ is the shortest path length between node i and node j in G, Gi is the efficiency of each vertex i.

***Modularity*** The modularity measure Q (p) for a given partition p of the human brain structural brain network is defined as:

Q(p) = $\sum_{s=1}^{M} [\frac{ks}{K}-(\frac{ds}{2K})^{2}]$

Where M is the number of modules, K is the number of connections in the network, ks is the number of connections between nodes in module s, and ds is the sum of the degrees of the nodes in modules.

***Nodal/edge betweenness*** The betweenness Bi of a node *i* is defined as the number of shortest paths between any two nodes that run through node i [^9^](#_ENREF_9), while the betweenness centrality of an edge is defined as the number of shortest paths between any 2 nodes in the network that pass through [^10^](#_ENREF_10).

**Tables**

| **Table S1. Demographic information for each group** | | |
| --- | --- | --- |
|  | **HC** (N=73) | **CD** (N=43) |
| Age | 15.6±0.84 | 14.6±1.11 |
| IQ | 107.89±7.15 | 99.24±10.03 |
| Gender (male/female) | 59/14 | 32/11 |

HC: healthy control, CD: conduct disorder.

| **Table S2. 34 labels (nodes) in brain network of each hemisphere.** | | | |
| --- | --- | --- | --- |
| **no.** | **labels** | **no.** | **lables** |
| 1 | bankssts_thickness | 18 | parsorbitalis_thickness |
| 2 | caudal ACC_thickness | 19 | parstriangularis_thickness |
| 3 | caudalmiddlefrontal_thickness | 20 | pericalcarine_thickness |
| 4 | cuneus_thickness | 21 | postcentral_thickness |
| 5 | entorhinal_thickness | 22 | posteriorcingulate_thickness |
| 6 | fusiform_thickness | 23 | precentral_thickness |
| 7 | inferiorparietal_thickness | 24 | precuneus_thickness |
| 8 | inferiortemporal_thickness | 25 | rostral ACC_thickness |
| 9 | isthmuscingulate_thickness | 26 | rostralmiddlefrontal_thickness |
| 10 | lateraloccipital_thickness | 27 | superiorfrontal_thickness |
| 11 | lateralorbitofrontal_thickness | 28 | superiorparietal_thickness |
| 12 | lingual_thickness | 29 | superiortemporal_thickness |
| 13 | medialorbitofrontal_thickness | 30 | supramarginal_thickness |
| 14 | middletemporal_thickness | 31 | frontalpole_thickness |
| 15 | parahippocampal_thickness | 32 | temporalpole_thickness |
| 16 | paracentral_thickness | 33 | transversetemporal_thickness |
| 17 | parsopercularis_thickness | 34 | insula_thickness |

*Note:* ACC, anterior cingulate.

**Table S3**

Clusters of cortical alterations in adolescents in both hemispheres (p<0.01, corrected)

| Cluster no. | Max | Size  (mm^2^) | TalX | TalY | TalZ | Number of  vertices | Annotation |
| --- | --- | --- | --- | --- | --- | --- | --- |
| left hemisphere | | | | | | | |
| 1 | -2.7212 | 633.69 | -12.9 | -96.8 | 14.5 | 845 | Lateral occipital |
| 2 | -2.0655 | 514.56 | -23.3 | 42.2 | -10.7 | 867 | lOFC |
| right hemisphere | | | | | | | |
| 1 | 4.0 | 2065.87 | 11.3 | 50.8 | 10.6 | 2969 | Superiorfrontal |
| 2  3  4 | -4.0  -4.0  -4.0 | 1636.60  1195.60  1018.63 | 65.2  30.6  61.5 | -17  -62.2  -34.9 | 3.0  -9.3  -14.2 | 3899  1986  1538 | Superior temporal  Fusiform  Middletemporal |

CD, conduct disorder; HC, healthy controls; lOFC, lateral orbitofrontal cortex; Max, log_10_ (p value); Tal (X, Y, Z), Talairach (X, Y, Z).

**Figures**


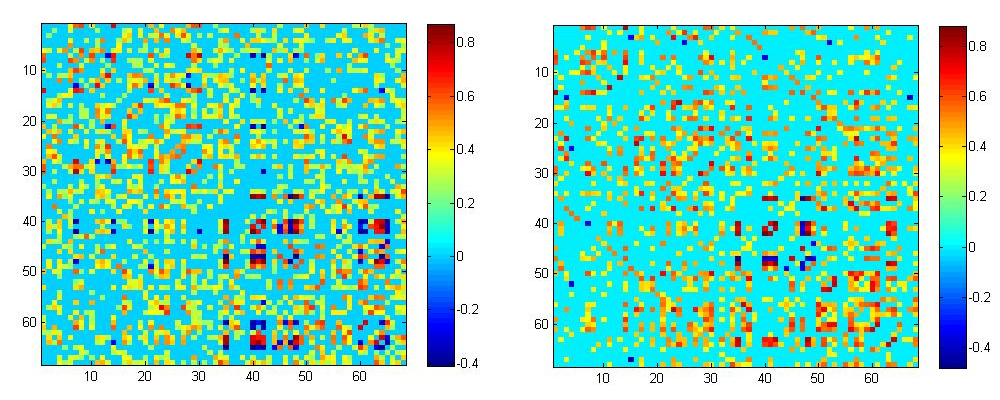


Figure S1. These two connection matrixes were derived from Pearson correlations of cortical thickness between 68 regions in HC (left) and CD (right) respectively. The label of each node was presented in Supplemental Table S2.


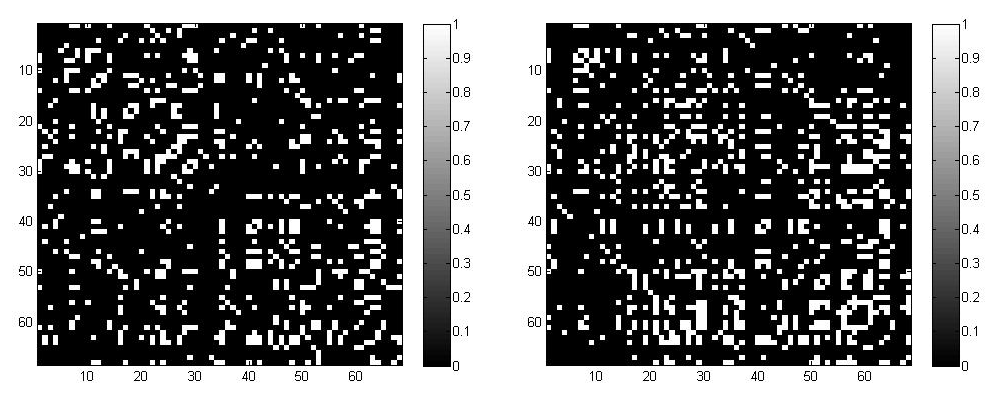


Figure S2. These two binary matrixes were constructed by thresholding the correlation matrix in HC (left) and CD (right) by FDR correction. Only the interregional pathways with strong correlations due to the conservative FDR threshold (Genovese et al. 2002) were included in this matrix. For the labels of these 68 regions, see Supplementary Table S2.

Supplemental References

1 Watts, D. J. & Strogatz, S. H. Collective dynamics of 'small-world' networks. *Nature* **393**, 440-442, doi:10.1038/30918 (1998).

2 Bernhardt, B. C., Chen, Z., He, Y., Evans, A. C. & Bernasconi, N. Graph-Theoretical Analysis Reveals Disrupted Small-World Organization of Cortical Thickness Correlation Networks in Temporal Lobe Epilepsy. *Cereb. Cortex* **21**, 2147-2157, doi:DOI 10.1093/cercor/bhq291 (2011).

3 He, Y., Chen, Z. & Evans, A. Structural insights into aberrant topological patterns of large-scale cortical networks in Alzheimer's disease. *J. Neurosci.* **28**, 4756-4766, doi:10.1523/JNEUROSCI.0141-08.2008 (2008).

4 Achard, S., Salvador, R., Whitcher, B., Suckling, J. & Bullmore, E. A resilient, low-frequency, small-world human brain functional network with highly connected association cortical hubs. *J. Neurosci.* **26**, 63-72, doi:10.1523/JNEUROSCI.3874-05.2006 (2006).

5 Wang, J. *et al.* GRETNA: a graph theoretical network analysis toolbox for imaging connectomics. *Front. Hum. Neurosci.* **9** (2015).

6 Latora, V. & Marchiori, M. Efficient behavior of small-world networks. *Phys. Rev. Lett.* **87**, 198701 (2001).

7 Newman, M. E. Modularity and community structure in networks. *Proc. Natl. Acad. Sci. U. S. A.* **103**, 8577-8582, doi:10.1073/pnas.0601602103 (2006).

8 Chen, Z. J., He, Y., Rosa-Neto, P., Germann, J. & Evans, A. C. Revealing modular architecture of human brain structural networks by using cortical thickness from MRI. *Cereb. Cortex* **18**, 2374-2381, doi:10.1093/cercor/bhn003 (2008).

9 Freeman, L. C. A set of measures of centrality based on betweenness. *Sociometry*, 35-41 (1977).

10 Girvan, M. & Newman, M. E. Community structure in social and biological networks. *Proc. Natl. Acad. Sci. U. S. A.* **99**, 7821-7826, doi:10.1073/pnas.122653799 (2002).
